# Release and Degradation Mechanism of Modified Polyvinyl Alcohol-Based Double-Layer Coated Controlled-Release Phosphate Fertilizer

**DOI:** 10.3390/polym16081041

**Published:** 2024-04-10

**Authors:** Teng Sun, Dekang Zhan, Xiangzhu Wang, Qingjie Guo, Mingzhou Wu, Pu Shen, Man Wu

**Affiliations:** 1 Laboratory of Green & Smart Chemical Engineering in Universities of Shandong, College of Chemical Engineering, Qingdao University of Science and Technology, Qingdao 266042, China; 13791692150@163.com (T.S.); zhan_dk@163.com (D.Z.); 13500736837@163.com (X.W.); qingjie_guo@163.com (Q.G.); 2 College of Health Science and Environmental Engineering, Shenzhen Technology University, Shenzhen 518118, China; wumingzhou@sztu.edu.cn; 3 Key Laboratory of Peanut Biology, Genetics & Breeding, Shandong Peanut Research Institute, Ministry of Agriculture and Rural Affairs, Shandong Academy of Agricultural Sciences, 126 Wannianquan Road, Qingdao 266100, China

**Keywords:** controlled release, phosphate fertilizers, biochar doping, biodegradability, double-layer film, polyvinyl alcohol

## Abstract

This study aims to improve the slow-release performance of a film material for a controlled-release fertilizer (CRF) while enhancing its biodegradability. A water-based biodegradable polymer material doped with biochar (BC) was prepared from modified polyvinyl alcohol (PVA) with polyvinylpyrrolidone (PVP) and chitosan (CTS), hereinafter referred to as PVA/PVP–CTS_a_BC_b_. An environmentally friendly novel controlled-release phosphate fertilizer (CRPF) was developed using PVA/PVP-CTS_8%_BC_7%_ as the film. The effect of the PVA/PVP-CTS_8%_BC_7%_ coating on the service life of the CRPF was investigated. The film was characterized via stress–strain testing, SEM, FTIR, XRD, and TGA analyses. The addition of the CTS modifier increased the stress of PVA/PVP-CTS_8%_ by 7.6% compared with that of PVA/PVP owing to the decrease in the crystallinity of PVP/PVP-CTS_8%_. The hydrophilic –OH groups were reduced due to the mixing of CTS and PVA/PVP. Meanwhile, the water resistance of the PVA/PVP-CTS_8%_BC_7%_ was improved. And the controlled-release service life of the CRPF was prolonged. Moreover, the addition of BC increased the crystallinity of the PVA/PVP-CTS_8%_ by 10%, reduced the fracture elongation of the material, and further improved the biodegradability of the PVA/PVP-CTS_8%_BC_7%_. When the amount of BC added was 7%, the phosphorus release rate of the CRPF was 30% on the 28th day. Moreover, the degradation rate of the PVA/PVP-CTS_8%_BC_7%_ polymer film was 35% after 120 days. This study provides basic data for applying water-based degradable polymer materials in CRFs.

## 1. Introduction

New advancements continue to occur in the field of fertilizers, aiming to provide nutrients essential for plant growth [1]. Phosphorus is an irreplaceable macronutrient for plant growth [2,3,4]. Most phosphorus fertilizers applied to soil systems are derived from rock phosphate, a nonrenewable resource with an estimated lifespan of 105–470 years [5]. Water-soluble phosphate fertilizers flow into water bodies through leaching and transport to groundwater and other means. This reduces the effective absorption of phosphorus by plants, causing environmental pollution and resource waste. Therefore, the controlled release of phosphorus from fertilizers can improve phosphorus use and prevent water and soil pollution [6,7].

Compared with traditional fertilizers, film-coated controlled-release fertilizers (CRFs) improve the utilization rate of nutrients in fertilizers by reducing environmental pollution and resource waste caused by nutrient loss. Coating traditional fertilizers with a film effectively slows down the release of nutrients and increases their utilization rate [8,9]. Research revealed that CRFs coated with synthetic polymers exhibit satisfactory controlled-release performance [10,11,12]. Lu et al. [13] used polyolefin wax-modified polyurethane to coat diammonium phosphate. The coating reduced the specific surface area of the fertilizer and improved the hardness to achieve enhanced controlled release. Zhao et al. [14] used sulfur-reinforced castor oil–modified polyurethane as a coating to enhance material strength and swelling performance, considerably increasing the controlled-release service life of urea to 210 days. However, refractory substances (such as polyurethane and polyester resin) may lead to undesirable accumulation of plastic residues in the soil. Soil degradation may occur when the accumulation of refractory substances exceeds 50 kg·ha^−1^ year^−1^ [12,15,16]. Therefore, these materials must be replaced by environmentally friendly polymers, particularly biodegradable polymers and recycled materials, to reduce environmental damage. Liu et al. [17] coated a phosphate fertilizer with waste cooking oil and modified its surface with carbon nanotubes to enhance the hydrophobicity of the film; the release cycle of the phosphate fertilizer reached more than 67 days. However, CRFs coated with natural polymer material film exhibit a short service life. Such CRFs cannot continuously provide nutrients for the entire growth cycle of crops. Thus, achieving a better controlled-release service life for fertilizers with reasonable biodegradability of the film material is essential. Accordingly, environmentally friendly and biodegradable organic polymer-coated materials with excellent controlled-release performance have attracted considerable research attention with respect to coated CRFs [18].

Polyvinyl alcohol (PVA) is an organic polymer and a nontoxic coating material [19]. However, PVA has poor water resistance and stress–strain properties and is degradable, limiting its application in CRFs [20,21]. Studies have revealed that the properties of PVA can be improved via modifications such as doping. Lum et al. [22] used boric acid to modify starch, increasing the crosslinking degree between starch and PVA. Improved water resistance enhanced the utilization of elemental nitrogen (N). However, high concentrations of boric acid may affect the physicochemical properties of soil. Polyvinyl pyrrolidone (PVP) exhibits good compatibility with PVA. Combining PVA and PVP with a material can improve its water resistance properties. Furthermore, both polymers are environmentally safe [23,24].

Chitosan (CTS), the second-largest natural biopolysaccharide, is insoluble in water and has good biocompatibility. PVA/CTS hydrogel is an ideal tissue scaffold in the medical field owing to its water retention and antibacterial properties [25]. Introducing CTS improves the stress–strain properties of PVA [26,27]. Further, rigid groups, such as –OH and -COOH groups, can be introduced through chemical crosslinking. Therefore, the mechanical properties of PVA films were improved by forming bonds between PVA and CTS [28].

Biochar (BC) has a porous structure that positively influences properties such as soil water holding capacity, pH, and ion exchange characteristics [29]. Many studies have demonstrated that introducing BC into polymer materials can improve the degradation rate of polymer materials in soil because BC can adsorb microorganisms, which can degrade polymer materials [30,31]. Kassem et al.’s [2] research has shown that methyl hydroxyethyl cellulose doped with BC can reduce the water absorption rate of the material. Biobased nanocomposite formulations comprising cellulosic BC are suitable for producing slow-release phosphate fertilizers with reduced phosphate leaching. Thus, BC exhibits considerable potential in developing film materials for excellent controlled-release performance.

This study focused on developing a green novel water-based biodegradable polymer material using biodegradable PVA and PVP with CTS and BC as film materials. A controlled-release phosphate fertilizer (CRPF) with a double-layer film was prepared using the water-based biodegradable polymer material as an outer coating. The effects of introducing CTS and BC on the properties and structure of PVA/PVP were investigated. Furthermore, the phosphate release behavior of the double-layer film-coated CRPF in water was investigated.

## 2. Materials and Methods

### 2.1. Materials Used in the Experiment

The water-based biodegradable polymer material were produced with the following materials: BC derived from orange peel; PVA (Sinopharm Chemical Reagent Co., Ltd., Shanghai, China, purity ≥ 99.0%, HU test); PVP K30 (Sinopharm Chemical Reagent Co., Ltd., GR, Wokai); CTS (Sinopharm Chemical Reagent Co., Ltd., BR, HU test); glacial acetic acid (Tianjin Fengfeng Ship Chemical Reagent Science and Technology Co., Ltd., Tianjin, China, AR); potassium dihydrogen phosphate (Tianjin Beichen Founder Reagent Factory, Tianjin, China, AR); ammonium metavanadate (Aladdin, Shanghai, China, AR); nitric acid (Tianjin Damao Chemical Reagent Factory, Tianjin, China, GR); ammonium molybdate (Sinopharm Group Chemical Reagent Co., Ltd., Shanghai, China, AR); bentonite; starch; phosphate fertilizer (diammonium phosphate particles).

### 2.2. Preparation of a Double-Layer CRPF

#### 2.2.1. Preparation of BC

The orange peels were cleaned and dried in an oven (Shanghai Longyue Instrument and Equipment Co., Ltd., Shanghai, China) at 50 °C for 24 h and pulverized using a pulverizer. These samples were transferred to a tube furnace and pyrolyzed at 300 °C, 500 °C, and 700 °C for 2 h in the presence of N_2_. BC was sieved using three meshes of size 0.074, 0.154, and 0.224 mm. Sieved BC was used to prepare subsequent PVA/PVP materials.

#### 2.2.2. Preparation of BC-Doped CTS-Modified PVA/PVP

A certain amount of PVA and deionized water were added to a three-necked flask and placed in an instantaneous thermostatic heating magnetic stirrer (Shanghai Lichen Gangxi Instrument Technology Co., Ltd., Shanghai, China). The temperature was increased to 95℃, and the mixture was stirred until complete dissolution of the PVA. Subsequently, the temperature was lowered and maintained at 60℃. Meanwhile, PVP was added with a mass of 1/3 of PVA and mixed thoroughly for 2 h to obtain PVA/PVP. Further, CTS was dissolved in an aqueous solution of glacial acetic acid. This solution was then added to PVA/PVP and mixed well for 1 h to obtain PVA/PVP-CTS_a_ (a: CTS content as a percentage of the PVA/PVP content). Finally, BC was added to this solution, which was then stirred for 1 h to obtain PVA/PVP-CTS_a_BC_b_ (b: BC content as a percentage of the PVA/PVP content).

#### 2.2.3. Preparation of the First and Second Layers of the Film Coating for the CRPF

Diammonium phosphate particles (DAPs) with a particle size of 3 mm were selected for this study. Bentonite clay and starch with a mass ratio of 20:1 (*w*/*w*) as the first layer of the film were added to the rotary drum coating machine at 50 °C to obtain a single layer of the coated fertilizer (BP). After the first-layer film coating, PVA/PVP-CTS_a_BC_b_ as the second-layer film was sprayed onto the surface of the fertilizer using an atomization nozzle at a pressure of 0.8 MPa. The rotary drum angle for coating was 45°, with a rotating speed of 50 r·min^−1^. In this way, double-layer CRPF (c-CRPF_d_; c: mass of second-layer film as a percentage of the mass of DAPs, d: types of second-layer films) was obtained.

### 2.3. Macroscopic Properties of Water-Based Biodegradable Polymer Material

#### 2.3.1. Water Absorbency of PVA/PVP, PVA/PVP-CTS_a_, and PVA/PVP-CTS_a_BC_b_

Each polymer film was cut into small pieces with dimensions of 5 cm × 5 cm. These specimens were immersed in deionized water for 3 h at room temperature. Excess water on the surface of the specimens was removed. Then the specimens were weighed. The mass was recorded as M_1_. Finally, the water-immersed film specimens were placed in an oven at 50 °C and dried to a constant weight. The mass of the dried film specimens was recorded as M_2_. The water absorption rate was calculated as follows:

A = M_1_/M_2_ × 100%.

#### 2.3.2. Biodegradability of PVA/PVP, PVA/PVP-CTS_8%_, and PVA/PVP-CTS_8%_BC_b_

The biodegradability of PVA/PVP, PVA/PVP-CTS_8%_, and PVA/PVP-CTS_8%_BC_b_ was determined using the soil burial method reported by Zou et al. [15]. The dried film material was cut into small pieces with dimensions of 5 cm × 5 cm and buried in soil in 20 mL sample tubes. Each film was set at nine sampling points. Three parallel experiments were set up for each sampling point. The specimens were buried in soil for 120 days. The specimens were removed from the soil in the sample tubes every few days. After sampling, the soil on the specimens was washed with deionized water. The washed specimens were oven-dried in an oven at 50 °C until they reached a constant weight. The rate of mass loss of the sample in the soil was calculated using the following formula:

D = (M_1_ − M_2_)/M_1_ × 100%

where D is the mass loss rate (%), M_1_ is the initial mass of the specimen before burial, and M_2_ is the remaining mass of the specimen after burial.

#### 2.3.3. Water Contact-Angle Test

The hydrophilicity of PVA/PVP, PVA/PVP-CTS_8%_, and PVA/PVP-CTS_8%_BC_7%_ was investigated using the sl200ks optical-contact-angle and interfacial-tension meter (Zhengzhou Kotai Experimental Equipment Co., Ltd., Zhengzhou, China). The microsyringe was adjusted to form a drop of water of 5 μm at the tip of the needle. The sample was placed horizontally close to the microsyringe to ensure proper contact between the sample surface and the droplet. The sample was removed, and the contact angle was measured after 60 s. The sample was tested thrice, and the average value of the measured contact angle was considered.

#### 2.3.4. Mechanical Performance Tests

The mechanical properties of the PVA/PVP, PVA/PVP-CTS_8%_, and PVA/PVP-CTS_8%_BC_7%_ series were investigated using a universal tensile machine (Zhengzhou Kotai Experimental Equipment Co., Ltd., Zhengzhou, China). First, the specimens were cut into dumbbell-shaped strips. The strips were ensured to be free of notches, uniformity, and air bubbles. Detailed data of the samples, such as thickness and width, were measured using a thickness gauge and a vernier caliper. The specimen was then clamped firmly and smoothly on the fixture. The sample was stretched at a tensile speed of 500 mm·min^−1^ until the sample broke and failed. The samples of each material were tested five times to obtain average values for tensile strength and stress strength.

### 2.4. Phosphorus Release Characteristics

The phosphorus release characteristics were determined by the methodology described by Kassem et al. [2]. The phosphorus release characteristics of the samples, DAPs, BP, CRPF, etc., were investigated in water at room temperature. P was denoted as the control sample. Approximately 10 g of each sample was placed in a 250 mL plastic bottle containing 200 mL deionized water. Leachate samples were collected on days 1, 3, 5, 7, 14, 21, 28, 35, 42, 49, 60, and 70 from each group of fertilizers to determine the phosphorus concentration using a photoelectric calorimeter. Phosphorus release was calculated based on absorbance measured at 425 nm. The service life of CRPF can be defined as the time required for the cumulative release rate of phosphorus to reach 80% of the total phosphorus.

### 2.5. Characterization of Water-Based Biodegradable Polymer Material

#### 2.5.1. Scanning Electron Microscopy

The surface morphology of the PVA/PVP-CTS_8%_BC_7%_ samples before and after the release test was tested using scanning electron microscopy (SU8600, HITACHI, Tokyo, Japan). The PVA/PVP-CTS_8%_BC_7%_ surface was sprayed with gold under vacuum; the acceleration voltage was 5.0 kV. The samples required for cross-sectional observation were subjected to brittle fracture by passing the films through liquid nitrogen before analysis.

#### 2.5.2. X-ray Diffraction

The crystallinity of the three samples, PVA/PVP, PVA/PVP-CTS_8%_, and PVA/PVP-CTS_8%_BC_7%_, was determined via X-ray diffraction. A fully automated multifunctional X-ray diffractometer with a scanning speed of 5°/min and a scanning range of 5°–80° was used for this analysis.

#### 2.5.3. Fourier Transform Infrared Spectroscopy

The chemical properties of the PVA/PVP and PVA/PVP-CTS_8%_BC_7%_ were characterized via Fourier transform infrared (FTIR) spectroscopy (Nicolet IS10, Waltham, MA, USA). A sample weighing 2 mg was placed in a ball mill. Then, 300 mg of dried potassium bromide powder was added, and the mixture was ground uniformly. Pressed tablets were prepared by weighing 250 mg and pressurizing at 15 MPa for 20 s. The surface functional groups of the sample were analyzed via FTIR spectroscopy at a scanning rate of 500–4000 cm^−1^.

#### 2.5.4. Thermal Properties

The thermodynamic stabilities of PVA/PVP, PVA/PVP-CTS_8%_, and PVA/PVP-CTS_8%_BC_7%_ were investigated by thermogravimetric analysis (TGA) using a NETZSCH STA 409PC Thermogravimetric Analyzer (NETZSCH Instruments Co., Ltd., Selb, Germany). A 10 mg sample was pyrolyzed from 50 °C to 500 °C under a nitrogen atmosphere at 10 °C min^−1^.

## 3. Results and Discussion

### 3.1. Effect of Biochar Addition on the Water Absorption of Water-Based Biodegradable Polymer Materials

**Figure 1:**
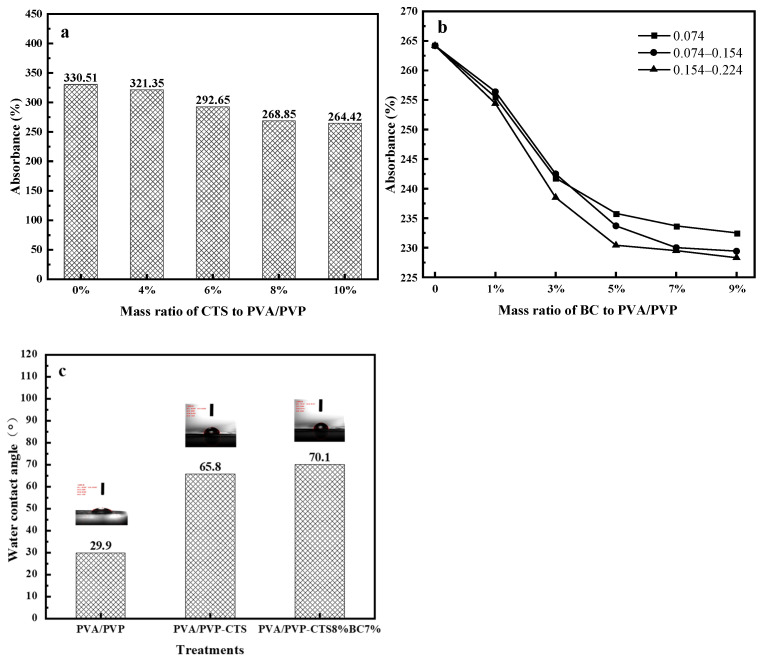
Water absorption of water-based biodegradable polymer materials: (**a**) water absorption of PVA/PVP-CTS_a_ with different concentrations of CTS; (**b**) water absorption of PVA/PVP- CTS_8%_BC_b_ doped with different BC particle sizes; (**c**) water contact angles.

Water absorption and degradability are essential for evaluating degradable biomaterials [32]. Figure 1a–c shows the water absorption values and water contact angles of the studied water-based polymer materials. The water absorption of PVA/PVP-CTS decreases with increasing CTS concentration. When the concentration of CTS reaches 8%, the water absorption of water-based biodegradable polymer materials tends to stabilize. PVA/PVP-CTS_8%_ was selected for BC doping in subsequent experiments.

The particle size and pyrolysis temperatures of different BCs also affect the water absorption of PVA/PVP-CTS_8%_BC_b_. A water-based biodegradable polymer material with low water absorption can be used for coating phosphate fertilizers. BC was prepared from orange peels at three different pyrolysis temperatures: 300 °C, 500 °C, and 700 °C. The water absorptions of BC were 179.23%, 220.65%, and 226.89% for the pyrolysis temperatures of 300 °C, 500 °C, and 700 °C, respectively. The water absorptions of BC gradually increased with increasing pyrolysis temperature. This can be attributed to the fact that the BET-specific surface area and microporous volume of BC increase with increasing pyrolysis temperature [33,34]. The larger specific surface area and well-developed pore structure provide more adsorption sites for water molecules [33].

Adding BC improves the hydrophobicity of PVA/PVP-CTS_8%_BC_b_ and reduces its water absorption rate. BC with a particle size of 0.154–0.224 mm exhibits a lower water absorption rate than BCs with other particle sizes. Further, the water absorption rate of BC is lower than that of PVA/PVP-CTS_8%_; thus, the higher the BC mass percentage, the lower the overall water absorption rate of the water-based biodegradable polymer materials. Once the BC content exceeds 7%, the water absorption rate of the PVA/PVP-CTS_8%_BC_b_ material stabilizes. The water contact-angle test revealed that the water contact angle of the PVA/PVP-CTS_8%_BC_7%_ material increased from 29.9° to 70.1° after introducing CTS and BC. This indicates that the hydrophobicity of the material improved. The lower the water absorption of the coating film, the better the slow-release performance of the fertilizer [35,36]. PVA/PVP-CTS_8%_ and BC particle sizes of 0.154–0.224 mm were selected for subsequent testing.

### 3.2. Fourier Transform Infrared Spectroscopy

**Figure 2:**
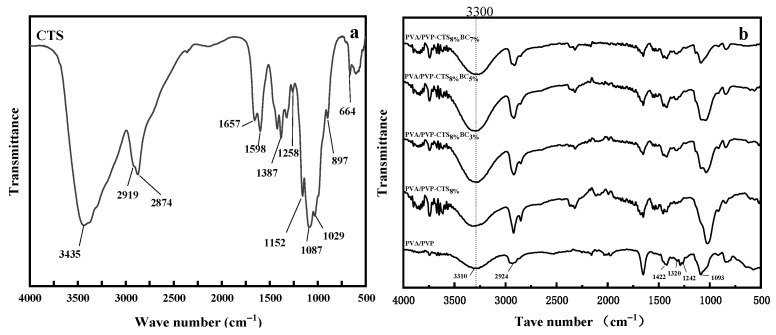
FTIR spectra of (**a**) CTS and (**b**) water-based biodegradable polymer materials.

In the infrared spectrum of CTS, as shown in Figure 2a, a prominent peak at 3435 cm^−1^ represents the overlapping stretching vibrations of ν (–OH) and ν (–NH_2_), indicating strong hydrogen bonding. The adjacent peaks at 2919 cm^−1^ and 2874 cm^−1^ correspond to ν (–CH_3_) and ν(–CH_2_), respectively, showcasing the presence of methylene and methyl groups. The intensity and sharpness of these peaks suggest a high degree of deacetylation in CTS. The characteristic absorption peak of the amide I band is at 1657 cm^−1^, while the deformation vibration absorption peak of δ is observed at 1598 cm^−1^. Additionally, a peak at δ (C–CH_2_) at 1387 cm^−1^ signifies the vibrational absorption of δ (–NH_2_), hinting at acetyl groups within the chitosan molecule [27]. The deformation vibration absorption peak at δ(–OH) is at 1258 cm^−1^, alongside the characteristic absorption peaks of β-glycosidic bonds at 1152 cm^−1^ and 897 cm^−1^, further delineating the CTS structure. The ν (C–O) vibration absorption peaks at 1087 cm^−1^ and 1029 cm^−1^ and the crystallization-sensitive peak at 664 cm^−1^ complete the CTS spectral profile. Conversely, the infrared spectrum of PVA/PVP, as shown in Figure 2b, expands the expected range of ν(–OH) stretching and stretching vibration peaks from 3200 cm^−1^ to 3700 cm^−1^, merging into the lower wavenumbers associated with methylene and methyl group vibration absorption peaks at 2924 cm^−1^. This broadening indicates extensive hydrogen bonds between molecular chains and molecules [21]. The deformation absorption peaks at 1422 cm^−1^ (δ(–CH_2_)) and 1320 cm^−1^ (δ(–OH)), along with the stretching vibration peak of ν (C-O) at 1090 cm^−1^, reinforce this observation. Comparing the PVP/PVP-CTS_8%_ infrared spectrum with pure CTS reveals that the integration of PVA/PVP causes the key peak of CTS at 3435 cm^−1^, corresponding to ν (–OH) and ν (–NH_2_), to widen and shift toward lower wavenumbers, indicating a strong hydrogen bonding interaction between CTS and PVA/PVP. This interaction induces shifts in functional groups. Notably, the deformation vibration absorption peak of δ (–OH) at 1258 cm^−1^ disappears owing to reduced –OH presence upon acetic acid addition. Similarly, the absorption peak of δ (–NH_2_) at 1598 cm^−1^ weakens with PVP/PVP doping, diminishing the –NH_2_ proportion. The characteristic absorption peaks of β-glycosidic bonds at 1152 cm^−1^ and 897 cm^−1^ in CTS are almost absent, and the crystallization sensitivity peak at 664 cm^−1^ is considerably weakened. This indicates that the blending with PVA/PVP disrupts the integrity of CTS molecular chains and reduces crystallinity [27]. The addition of different proportions of bacterial cellulose yields similar infrared spectra across the three blend components without introducing new absorption peaks. This suggests a physical mix of the components. However, the formation of hydrogen bonds between PVA/PVP and CTS molecules results in a tight bond, reducing the hydrophilic –OH group and enhancing the hydrophobicity of the material.

### 3.3. X-ray Diffraction Analysis

**Figure 3:**
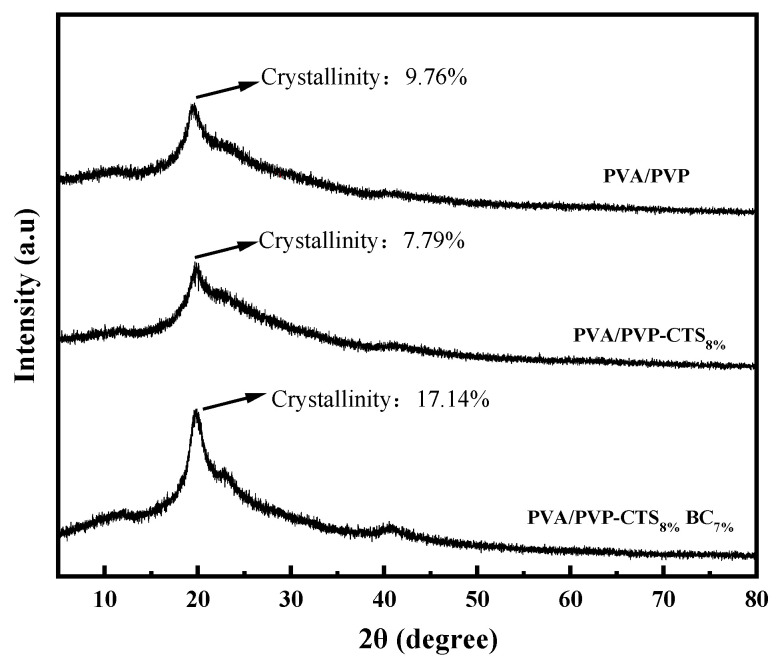
X-ray diffraction spectra of PVA/PVP, PVA/PVP-CTS_8%_, and PVA/PVP-CTS_8%_BC_7%_.

Figure 3 shows the XRD patterns of PVA/PVP, PVA/PVP-CTS_8%_, and PVA/PVP-CTS_8%_BC_7%_. The crystallinity of the materials was obtained using the area integration method. The crystallinity of the PVA/PVP material before the addition of CTS is 9.76%. The crystallinity of PVA/PVP-CTS_8%_ decreases to 7.79% after the addition of 8% CTS. This is consistent with the FTIR characterization results. The crystallinity of the PVA/PVP-CTS_8%_BC_7%_ material doped with 7% BC is 17.14%. The crystallinity of the PVP/PVP-CTS_8%_ material with CTS modification decreases. The crystallinity of the PVA/PVP-CTS_8%_BC_7%_ material increases after doping with BC. Additionally, the crystalline zones within the material are tightly arranged, increasing the material resistance to solvents and water [28]. However, the toughness of the material worsens. The PVA/PVP-CTS_8%_BC_7%_ easily broke during degradation, increasing the surface area in contact with the soil. Despite this, the PVA/PVP-CTS_8%_BC_7%_ demonstrates excellent degradability and environmental friendliness, making it suitable as a coating film for CRF.

### 3.4. Thermogravimetric Analysis

**Figure 4:**
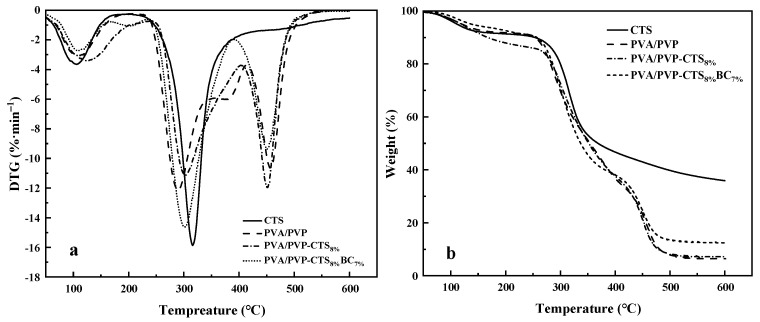
DTG (**a**) and TGA (**b**) of CTS, PVA/PVP, PVA/PVP-CTS_8%_, and PVA/PVP-CTS_8%_BC_7%_.

Figure 4a,b show the DTG and TGA curves of CTS, PVA/PVP, PVA/PVP-CTS_8%_, and PVA/PVP-CTS_8%_BC_7%_. Figure 4a demonstrates that the thermal degradation of CTS occurs in two stages. The first stage is from 50 °C to 150 °C. The thermal degradation efficiency is small in this interval, with a peak value of 3.64%·min^−1^ because of the bound water in CTS. The second stage is from 250 °C to 400 °C. The thermal degradation efficiency is the largest in this temperature range, with a peak value of 15.79%·min^−1^. The total mass fraction of weight loss is 64.09%. Thermal degradation of PVA/PVP film occurs in three stages. The first stage is from 50 °C to 150 °C, mainly for the evaporation of bound water. The second stage is from 250 °C to 350 °C, with a maximum weight loss rate of 12.09%·min^−1^, mainly corresponding to the breakage of the side chains of PVA and PVP and the formation of carbon build-up. The third stage is from 400 °C to 550 °C, where the thermal decomposition of PVA/PVP decreases and stabilizes. The main function of this stage is the thermal degradation of carbon deposited on the film. The CTS content in PVA/PVP caused the multiple peaks to overlap near the peak of the maximum weight loss rate. The maximum weight loss rate was 11.94%·min^−1^. The total degradation rate of PVA/PVP-CTS_8%_ increased from 93.29% to 92.42%. The hydrogen bonding between CTS and PVA/PVP enhanced the thermal stability of the film. Introducing BC resulted in a total film degradation rate of 83.32%, improving the overall thermal stability of the film. In addition, BC improved the thermal stability of PVA/PVP-CTS_8%_BC_7%_.

### 3.5. Mechanical Properties

**Figure 5:**
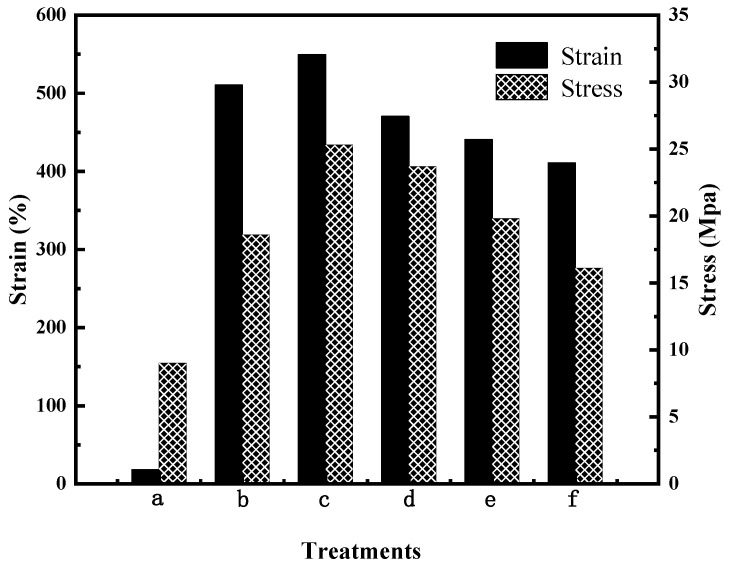
Mechanical properties of water-based biodegradable polymer materials: (a) CTS, (b) PVA/PVP, (c) PVA/PVP-CTS_8%_, (d) PVA/PVP-CTS_8%_BC_3%_, (e) PVA/PVP-CTS_8%_BC_5%_, and (f) PVA/PVP-CTS_8%_BC_7%_.

The mechanical properties of the PVA/PVP film before and after modification were investigated by tensile shear experiments. Mechanical properties are important characteristics for the films of CRPFs. Figure 5 shows that the tensile strength of the PVA/PVP film before modification is 18.6 MPa. Meanwhile, the corresponding elongation at break is 510.6%. The tensile strength of the CTS is 9 Mpa, and the elongation at break is 18%. After modification with 8% (wt) CTS, the tensile strength of the PVA/PVP-CTS_8%_ material is 25.3 MPa, and the elongation at break is 549.6%. After introducing CTS and BC, the elongation at break and the tensile strength of the PVA/PVP-CTS_8%_BC material are reduced. With an increase in BC content, the elongation at break of the PVA/PVP-CTS_8%_BC_b_ material is further reduced. The tensile strength of PVA/PVP-CTS_8%_BC_b_ is reduced from 23.7 MPa to 16.1 MPa by increasing the BC content from 3% to 7%. This is also related to the increase in crystallinity. The macromolecules of the PVA material are in a curled-up state without external force. In the event of an external force, the macromolecules are pulled until they break, resulting in a larger elongation at break and a higher stress. Owing to the addition of CTS in PVA/PVP, hydrogen bonds formed between CTS and PVA/PVP, resulting in a denser binding. However, introducing BC, which has a greater stiffness compared to PVA, destroys the coiled structure of the PVA macromolecules, thereby increasing brittleness and resulting in lower elongation at break [23]. The tensile strength of the CTS is 9 Mpa, and the elongation at break is 18%. The appropriate mechanical strength of the film material is conducive to the slow release of phosphate in CRPF. A higher elongation at break of the outer film can reduce the occurrence of rupture during CRPF controlled-release testing.

### 3.6. Effect of BC on the Degradability of PVA/PVP-CTS_8%_BC_b_

**Figure 6:**
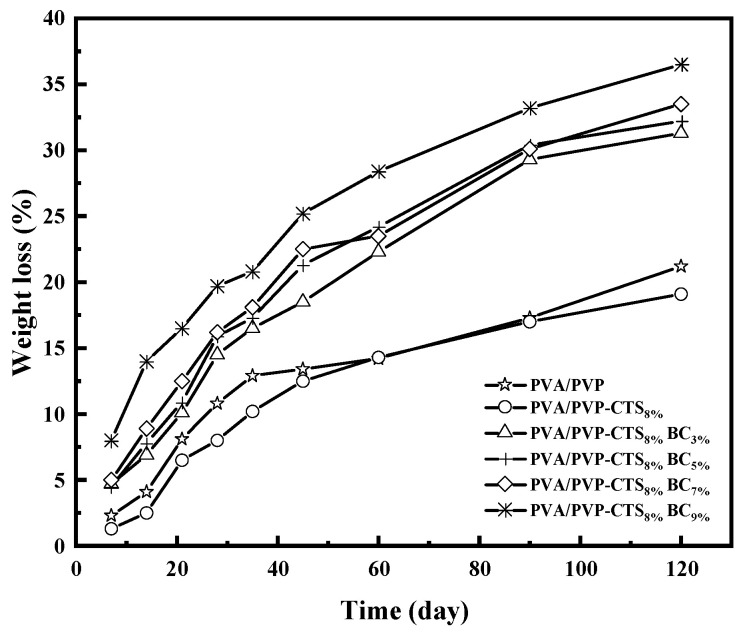
Mass loss rate of different amounts of BC doped with PVA/PVP-CTS_8%_ at different burial times.

The degradation of CRF coating films is the key to determining their environmental friendliness. Figure 6 shows the degradability of the studied polymers. The better the degradability, the better the environmental friendliness. Soil burial experiments provide a realistic degradation environment. PVA/PVP and PVA/PVP-CTS_8%_ exhibited a fast degradation rate in the first 28 days, followed by a gradual degradation rate after 45 days. Specifically, the degradation rate of PVA/PVP was 21.2% at 120 days, which decreased to 19.1% with the addition of CTS. The degradability further improved when BC with a particle size of 0.154–0.224 mm was introduced. Increasing the amount of BC added from 3% to 9% increased the degradation rate of the film from 31.3% to 36.5%. Introducing BC reduced the stress–strain mechanical properties, causing the film to be more susceptible to rupture; the BC provided a relatively alkaline environment for PVA/PVP-CTS_8%_, favoring its degradation in soil [37]. Moreover, BC provided attachment sites for microorganisms. The biodegradability of PVA/PVP-CTS_8%_BC_b_ film materials further increased due to microorganisms and bacterial flora, facilitating PVA decomposition in soil [23,38].

### 3.7. Double-Layer CRPF Structure and Phosphorus Release Characteristics in Water

**Figure 7:**
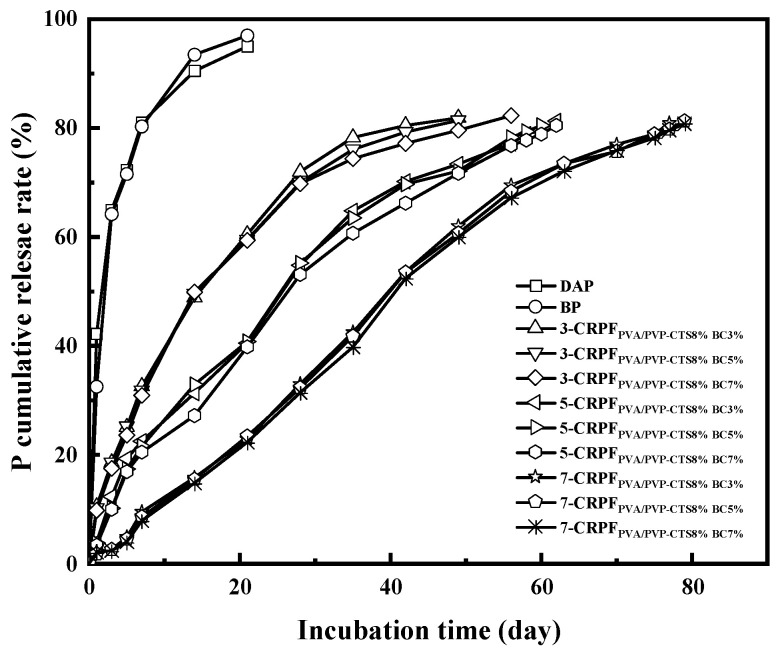
Cumulative phosphate release rate of fertilizers with different coating ratios at room temperature.

Figure 7 shows the phosphorus release rate and controlled-release service life of DAPs, BP, 3-CRPF_PVA/PVP-CTS8%BCb_, 5-CRPF_PVA/PVP-CTS8%BCb_, and 7-CRPF_PVA/PVP-CTS8%BCb_ in water. DAPs and BP showed a fast release rate for phosphate and a controlled-release service life of only 7 days. The controlled-release service life increased vastly using double-layer films. Different amounts and types of the second film showed varied effects on the controlled-release service life of the CRPF. For example, in the case of 3-CRPF_PVA/PVP-CTS8%BCb_, increasing the BC amount from 3% to 7% in the film increased the controlled-release service life from 49 to 56 days. The service life of CRPFs increases significantly with the increasing of second-layer film amounts. The average controlled-release service life of the CRPF increased from 50 to 80 days as the amount of the second-layer film increased from 3% to 7% (wt).

The effect of varying the amount of the second-layer film was greater than that of varying the amount of BC doping. Compared with the service life of DAPs and BP, the service life of the CRPFs was considerably increased. The outer PVA/PVP-CTS_8%_BC_b_ film slowed the entry of water molecules, releasing phosphorus gradually. Meanwhile, the PVA/PVP-CTS_8%_BC_b_ film was not easily damaged in water and had a high elongation at break. This resulted in an extension of the service life of the CRPFs. Introducing BC elevated the hydrophobicity of the PVA/PVP-CTS_8%_BC_b_ film, reducing phosphate dissolution. Upon increasing the content of the outer film material, the controlled-release service life of phosphorus first increased and then stabilized. Table 1 shows the controlled-release performance and soil degradation rate of the slow-release fertilizers using different coating materials. Compared with existing research, this study balances better controlled-release performance and biodegradability.

**Figure 8:**
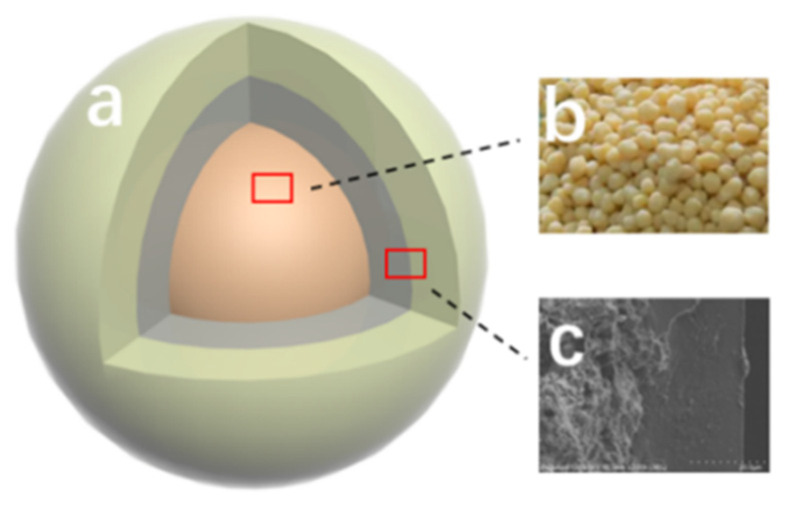
Structure of the double-layer controlled-release phosphate fertilizer: (**a**) Outer layer material; (**b**) Diammonium phosphate; (**c**) Inner layer material.

Figure 8 shows the structure of the double-layer CRPF. Fertilizer core-b is a phosphate fertilizer granule containing diammonium phosphate. The inner layer is wrapped with bentonite/starch as a 20:1 liner. The outer layer is PVA/PVP-CTS_8%_BC_b_ material. The inner layer acts as a cushioning material without a slow-release capability. The layer gradually absorbs water and dissolves. This is closely followed by fertilizer core water absorption and collapse. The increased contact between the water and fertilizer core accelerates the dissolution rate of fertilizer, resulting in a short controlled-release service life. Adding the inner layer fills the gap between the fertilizer core and the outer layer with the water-absorbing fluffy material. Therefore, the controlled-release fertilizer is supported by the outer layer without breaking. Meanwhile, the phosphate loss slows down. In this way, the controlled-release service life of the doubled-layer CRPF is extended.

### 3.8. SEM Images of CRPF Surface

**Figure 9:**
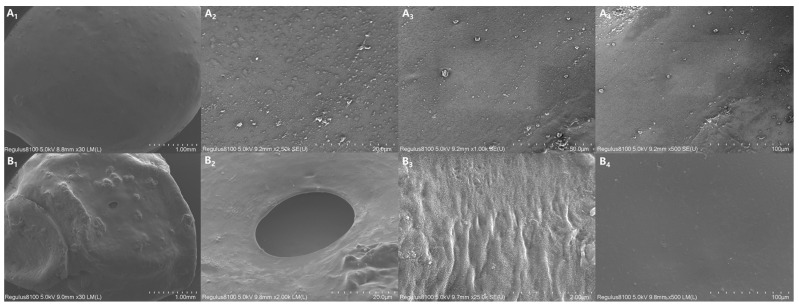
SEM images of the CRPF surface before and after the controlled-release test: (**A_1_**–**A_4_**) The surface of CRPF before the controlled-release test; (**B_1_**–**B_4_**) The surface of CRPF after the controlled-release test.

Figure 9 shows the SEM images of the surface of 7-CRPF _PVA/PVP-CTS8%BC7%_ before and after the controlled-release test. Before the controlled-release test of 7-CRPF _PVA/PVP-CTS8%BC7%_ (Figure 9(A_1_–A_4_)), the film of the CRPF surface was uniformly coated, with no vast cracks or holes observed. This indicated that the PVA/PVP-CTS_8%_BC_7%_ material could be completely coated on the surface of the CRPF core. The film forms a barrier, slowing the entry of water molecules into the CRPF core. This is conducive to preventing a large influx of water and slowing the release of phosphate from the coating during the early stages of the CRPF controlled-release test. Figure 9(B_1_–B_4_) shows the surface of 7-CRPF_PVA/PVP-CTS8%BC7%_ after the controlled-release test. The PVA/PVP-CTS_8%_BC_7%_ coating was intact, with no significantly damaged area or depression. Only small holes or wrinkles without rupture after the CRPF controlled-release test were observed. Due to the loss of the CRPF core during the CRPF controlled-release test, when the water absorption and dissolution of the film were more significant than the rupture elongation limit of the material, a few small holes were caused by film subsidence after drying. However, because of the double-layer structure, the inside of the CRPF did not directly break and dissolve. This indicated that the swelling performance of the material is excellent and that introducing CTS results in a denser film [18]. Therefore, the film cannot be ruptured extensively due to swelling during the CRPF controlled-release test. This is beneficial for releasing phosphorus from the CRPF.

## 4. Conclusions

A green water-based biodegradable polymer material was prepared via a blending reaction to encapsulate phosphate fertilizer. After optimizing the pyrolysis temperature, particle size, and amount of BC added, BC was selected with a pyrolysis temperature of 300 °C and a particle size range of 0.174–0.224 mm to produce PVA/PVP-CTS_8%_BC_7%_ film materials. Owing to hydrogen bonding between CTS and PVA/PVP, the density of the film increased and its –OH content decreased, improving the hydrophobicity of PVA/PVP-CTS_8%_. When 8% (*w*/*w*) CTS was added, the water absorption rate reduced to 268.85%, while the water contact angle increased to 68.5°.

With the addition of BC, the water absorption of the material was further reduced. The water absorption rate of PVA/PVP-CTS_8%_BC_7%_ reduced to 228%. Moreover, BC reduced the stress on PVA/PVP-CTS_8%_BC_b_. When the BC addition amount is 7%, the stress of PVA/PVP-CTS_8%_BC_7%_ reduces to 16.1 MPa. The crystallinity of the film is 8%, which is beneficial for improving degradation performance. The PVA/PVP-CTS_8%_BC_7%_ material can be degraded up to 35% in soil after 120 days. Moreover, a series of film materials exhibited good thermal stability. SEM analysis results show that PVA/PVP-CTS_8%_BC_7%_ can be entirely and uniformly coated on the fertilizer surface. The fertilizer does not extensively rupture after 120 days. The use of the environmentally friendly water-based biodegradable polymer material PVA/PVP-CTS_8%_BC_7%_ as a coating material exhibits considerable technical potential and wide applications in controlling phosphate release to improve the controlled-release service life and biodegradability of CRFs.

## Figures and Tables

**Table 1:** Controlled-release performance and degradation of different film materials.

Method	Controlled-Release Performance (Accumulated Release Amount/Time)	Degradation Performance (Accumulated Degradation Amount/Time)	Reference
Polyvinyl alcohol/polyvinylpyrrolidone/biochar	60%/20 days	30%/120 days	Chen et al. [23]
Starch–g-polyacrylic acid/natural rubber/polyvinyl alcohol	62%/3 days	90%/120 days	Tanan et al. [19]
Epoxy resin/biobased polyurethane	10%/20 days	8%/120 days	Liu et al. [18]
PVA/PVP-CTS_8%_BC_7%_	23%/21 days	35%/120 days	This work

## Data Availability

Data will be made available on request.
